# Formulae for Nutrient Enema

**Published:** 1915-02

**Authors:** 


**Formulae for Nutrient Enema.** The following list is useful as it gives a sufficient variety for choice. Nutrient enemas are difficult to compose extemporaneously:

1. Normal salt solution 7 : 1000.
2. Glucose solution 10 : 100.
3. Wine or cognac 5 or 10 : 100.
4. Brandy and glucose:

℞ Old cognac .......................... a teaspoonful
Glucose .................................. 20.0 gm.
Distilled water ............................ 200 c.c.

OR

℞ Glucose .................................. 20.00
Red wine .................................. 80.00
Distilled water ............................ 200.00

5. Proteids, fats, etc.

℞ Milk ...................................... 250.0

Two yolks of eggs.

Flour ...................................... 20.0

Red wine .................................. 15.0

Sydenham's laudanum, 4 drops.

Salt ............................................3.0

MIX.

℞ Peptone ............................................5.0
Two yolks of eggs.
Flour ............................................15.0
Wine ............................................60.0
Solution of dextrin, 20% (boiled and cooled) ....ad 300.0

℞ Sydenham's laudanum ..........................gtt iii
Pepsin ............................................0.50
Salt ............................................1.00
Two yolks of eggs.
Solution of peptone ....................2 tablespoonfuls
Solution of glucose, 10% ..........................100.00

℞ Laudanum ............................................3 drops
Sodium bicarbonate ............................................0.3
Sat. solution meat peptone ............................60.0
Water ............................................250.0

Le Gendre and Martinet, "Revue de Pharmacol. Med.," 1914, page 22.

